# Supplementary material for: Evidence of multiple genome duplication events in Mytilus evolution
Source: BMC Genomics. 2022 May 2;23:340. doi: 10.1186/s12864-022-08575-9 (PMC9063065; doi:10.1186/s12864-022-08575-9)
Supplement: Supplementary file 1 — Additional file 1: Figure S1. The k-mer distribution used for the estimation of genome size. The heterozygous and homozygous peaks of k-mer depth are clearly markers, suggesting a high-complexity genome. A The 17-mer distribution. Predicted genome size, 1,010,184,781 nt; B The 23-mer distribution. Predicted genome size, 1,096,306,163 nt. Table S1. Sequencing data, summary statistics. _ Estimation based on M. galloprovincialis and M. coruscus genomes size. Table S2. RepeatMasker statistics. _ repeats fragmented by insertions or deletions have been counted as one element. y LTR Finder results: 255,413 LTR pairs over 4932 regions and 151,703,353 bp. Table S3. Summary of annotation results for M. edulis gene models using a range of databases. _InterPro covers 12 databases (CDD-3.17,Coils-2.2.1, Gene3D-4.2.0, Hamap-2020 01, MobiDBLite-2.0, PANTHER-14.1, PRINTS-42.0, ProSitePatterns-2019 11, ProSitePro_les-2019 11, SFLD-4, SMART-7.1, SUPERFAMILY-1.75, TIGRFAM-15.0). Table S4. Mytilinae (subfamily) mitochondrial genomes. Table S5. Bivalvia (class) genome where Ka & Ks estimations were possible: All exhibit evidences of _-WGD and _-WGD. _ this study. Table S6. Bivalvia (class) genome and availability of gene models and annotations. _ this study. Table S7. Genes involved in immunity, stress response and shell formation under positive selection in M. galloprovincialis, M. edulis and M. coruscus. [file 12864_2022_8575_MOESM1_ESM.pdf]

RESEARCH

Evidence of multiple genome duplication events in *Mytilus* evolution

Ana Corrochano-Fraile, Andrew Davie, Stefano Carboni* and Michaël Bekaert

*Correspondence:
stefano.carboni@stir.ac.uk
Institute of Aquaculture, Faculty of Natural Sciences, University of Stirling, Stirling, FK9 4LA UK
Full list of author information is available at the end of the article

Supplementary Figure

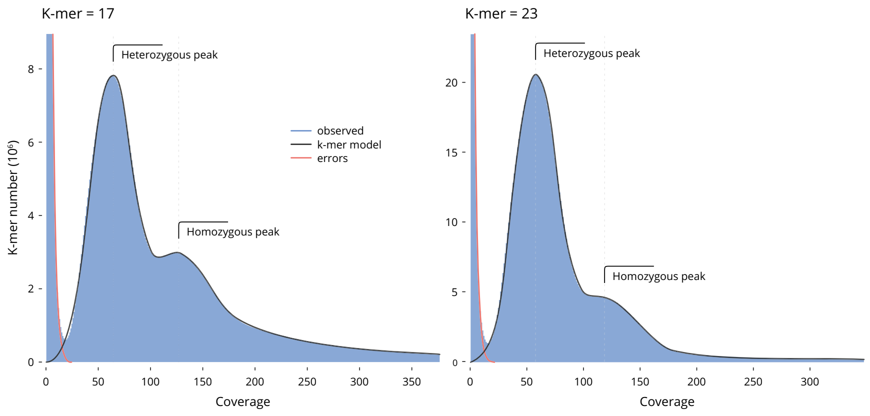

Figure S1 The k-mer distribution used for the estimation of genome size. The heterozygous and homozygous peaks of k-mer depth are clearly markers, suggesting a high-complexity genome. **A** The 17-mer distribution. Predicted genome size, 1,010,184,781 nt; **B** The 23-mer distribution. Predicted genome size, 1,096,306,163 nt.

Supplementary Tables

Table S1 Sequencing data, summary statistics. ∗ Estimation based on *M. galloprovincialis* and *M. coruscus* genomes size.

Category	Number/length
Total number of long reads	15,945,130
Total number of bases	111,654,433,463
Mean length	7,002 nt
Maximum read length	672,255 nt
Coverage*	64x
Total number of PE short reads	652,465,784
Total number of bases	195,739,735,200
Read length	150 nt
Coverage*	113x
Total number of RNA-seq PE short reads	50,593,080
Total number of bases	15,177,924,000
Read length	150 nt
Coverage*	9x

Table S2 RepeatMasker statistics. ∗ repeats fragmented by insertions or deletions have been counted as one element. † LTR_Finder results: 255,413 LTR pairs over 4,932 regions and 151,703,353 bp.

Element	Number of elements*	Length occupied	Percentage of sequence
SINEs	10,261	2,030,467 bp	0.11%
ALUs	0	0 bp	0.00%
MIRs	0	0 bp	0.00%
LINEs	343,643	130,980,160 bp	7.17%
LINE1	11,902	5,537,561 bp	0.30%
LINE2	7,970	3,762,443 bp	0.21%
L3/CR1	6,535	4,167,698 bp	0.23%
LTR elements†	4,932	151,703,353 bp	8.30%
DNA elements	99,267	25,504,172 bp	1.40%
hAT-Charlie	2,603	433,449 bp	0.02%
TcMar-Tigger	0	0 bp	0.00%
Unclassified	2,976,814	707,039,397 bp	38.70%
Small RNA	133	25,717 bp	0.00%
Satellites	2,252	400,532 bp	0.02%
Simple repeats	224,100	10,280,290 bp	0.56%
Low complexity	50,703	2,473,133 bp	0.14%
Total repeats		1,029,206,554 bp	56.33%

Table S3 Summary of annotation results for *M. edulis* gene models using a range of databases. ∗ InterPro covers 12 databases (CDD-3.17,Coils-2.2.1, Gene3D-4.2.0, Hamap-2020_01, MobiDBLite-2.0, PANTHER-14.1, PRINTS-42.0, ProSitePatterns-2019_11, ProSiteProfiles-2019_11, SFLD-4, SMART-7.1, SUPERFAMILY-1.75, TIGRFAM-15.0).

Database	Number annotated
PfamA	61,453
InterPro*	48,772
SwissProt	11,211
KEGG	51,091
GO	31,620
All	9,089
Total	69,246

Table S4 Mytilinae (subfamily) mitochondrial genomes.

Accession	Genome size	Species name	Common name
NC_018362.1	16,014 bp	*Perna viridis*	Asian green mussel
NC_044131.1	16,253 bp	*Septifer bilocularis*	-
NC_044128.1	17,582 bp	*Crenomytilus grayanus*	-
NC_030633.1	16,765 bp	*Mytilus chilensis*	Chilean mussel
NC_028706.1	18,145 bp	*Limnoperna fortunei*	Golden mussel
NC_026288.1	18,415 bp	*Perna perna*	Brown mussel
NC_024733.1	16,642 bp	*Mytilus coruscus*	Hard-shell mussel
NC_006886.2	16,744 bp	*Mytilus galloprovincialis*	Mediterranean mussel
NC_006161.1	16,740 bp	*Mytilus edulis*	Blue mussel
NC_007687.1	18,652 bp	*Mytilus trossulus*	Bay mussel
NC_015993.1	16,730 bp	*Mytilus californianus*	California mussel

Table S5 Bivalvia (class) genome where Ka & Ks estimations were possible: All exhibit evidences of α-WGD and β-WGD. $*$ this study.

Species	Peak/Mean (α-WGD)	Std. dev. (α-WGD)	Peak/Mean (β-WGD)	Std. dev. (β-WGD)
*M. edulis**	0.6132	0.1939	1.8196	0.7103
M. galloprovincialis	0.6010	0.1934	1.8109	0.7011
M. coruscus	0.5635	0.1996	1.7977	0.7082
C. gigas	0.6299	0.3699	1.6350	0.4601
C. virginica	0.4787	0.4921	1.7627	0.3479
M. yessoensis	0.6612	0.2278	1.7064	0.4722
P. maximus	0.6392	0.2759	1.8794	0.4541

Table S6 Bivalvia (class) genome and availability of gene models and annotations. $*$ this study.

Assembly	Reference	Species name	Common name	Gene Models
GCA_014843695.1	ASM1484369v1	*Archivesica marissinica*	Deep-sea clam	-
GCA_004382765.1	QAU_Acon_1.1	*Argopecten irradians* concentricus	Bay scallop	-
GCA_004382745.1	QAU_Airr_1.1	*Argopecten irradians* irradians	Bay scallop	-
GCA_002080005.1	Bpl_v1.0	*Bathymodiolus platifrons*	Cold seep mussel	-
GCA_001632725.1	ASM163272v1	*Corbicula fluminea*	Asian clam	-
GCF_902806645.1	cgigas_uk_roslin_v1	*Crassostrea gigas*	Pacific oyster	yes
GCA_005518195.2	NWPU_Cgig_v2	*Crassostrea gigas*	Pacific oyster	-
GCF_000297895.1	oyster_v9	*Crassostrea gigas*	Pacific oyster	-
GCA_000297895.2	ASM29789v2	*Crassostrea gigas*	Pacific oyster	-
GCA_011032805.1	ASM1103280v1	*Crassostrea gigas*	Pacific oyster	-
GCA_015776775.1	ASM1577677v1	*Crassostrea hongkongensis*	Hong Kong oyster	-
GCA_002022765.1	C_virginica_1.0	*Crassostrea virginica*	Eastern oyster	-
GCA_002022765.3	C_virginica-2.0	*Crassostrea virginica*	Eastern oyster	-
GCF_002022765.2	C_virginica-3.0	*Crassostrea virginica*	Eastern oyster	yes
GCA_012932295.1	ASM1293229v1	*Cyclina sinensis*	Venus clam	-
GCA_000806325.1	ASM80632v1	*Dreissena polymorpha*	Zebra mussel	-
GCA_007657795.1	UV_Dro_v1.1	*Dreissena rostriformis*	Quagga mussel	-
GCA_003130415.1	ASM313041v1	*Limnoperna fortunei*	Golden mussel	-
GCA_008271625.1	LuRhyn_1.0	*Lutraria rhynchaena*	Snout Otter Clam	-
GCA_016163765.1	CUHK_oyster_2.0	*Magallana hongkongensis*	Hong Kong oyster	-
GCA_015947965.1	ASM1594796v1	*Margaritifera margaritifera*	Freshwater pearlshell mussel	-
GCA_016617855.1	ASM1661785v1	*Megalonaias nervosa*	Washboard	-
GCA_014805675.1	ASM1480567v1	*Mercenaria mercenaria*	Northern quahog	-
GCF_002113885.1	ASM211388v2	*Mizuhopecten yessoensis*	Yesso scallop	yes
GCA_002113885.1	ASM211388v1	*Mizuhopecten yessoensis*	Yesso scallop	-
GCA_002080025.1	Mph_v1.0	*Modiolus philippinarum*	Philippine horse mussel	-
GCA_017311375.1	Mcoruscus_HiC	*Mytilus coruscus*	Hard-shell mussel	-
GCA_011752425.2	MCOR1.1	*Mytilus coruscus*	Hard-shell mussel	yes
GCA_011752425.1	MCOR1	*Mytilus coruscus*	Hard-shell mussel	-
GCA_905397895.1*	MEDL1	*Mytilus edulis*	Blue mussel	yes
GCA_019925275.1	PEIMSO	*Mytilus edulis*	Blue mussel	-
GCA_900618805.1	MGAL_10	*Mytilus galloprovincialis*	Mediterranean mussel	yes
GCA_000715055.1	mussel1.0	*Mytilus galloprovincialis*	Mediterranean mussel	-
GCA_001676915.1	ASM167691v1	*Mytilus galloprovincialis*	Mediterranean mussel	yes
GCA_903981925.1	v081	*Ostrea lurida*	Olympia oyster	-
GCA_902825435.1	PGEN-v1.0	*Panopea generosa*	Pacific geoduck	-
GCF_902652985.1	xPecMax1.1	*Pecten maximus*	King scallop	yes
GCA_002216045.1	PinMar1.0	*Pinctada imbricata*	Akoya pearl oyster	-
GCA_016161895.1	ASM1616189v1	*Pinna nobilis*	Noble penshell	-
GCA_016746295.1	UT_Pstr_1.0	*Potamilus streckersoni*	Brazos heelsplitter	-
GCA_009026015.1	ASM902601v1	*Ruditapes philippinarum*	Manila clam	-
GCA_003671525.1	Sgl1.0	*Saccostrea glomerata*	Sydney rock oyster	-
GCA_007844125.1	ASM784412v1	*Sinonovacula constricta*	Chinese razor clam	-
GCA_009762815.1	ASM976281v1	*Sinonovacula constricta*	Chinese razor clam	-
GCA_013375625.1	ASM1337562v1	*Tegillarca granosa*	Blood clam	-
GCA_003401595.1	ASM340159v1	*Venustaconcha ellipsiformis*	Ellipse	-

Table S7 Genes involved in immunity, stress response and shell formation under positive selection in *M. galloprovincialis*, *M. edulis* and *M. coruscus*.

Species	Category	ClusterID	Gene Names / Function (Ortholog cluster)	Ka/Ks	Ortholog reference
M. galloprovincialis	Immunity	9813	NIT1<br>Potential tumour suppressor in tumour progression	2.5	[1]
M. galloprovincialis	Stress	6307	Arylesterase / Paraoxonase<br>Confer resistance to organophosphate toxicity	1.4	[2]
M. galloprovincialis	Stress	1976	Serine/threonine kinase 17<br>Cellular processes, proliferation, apoptosis, and differentiation. Abiotic stress	1.4	[3]
M. galloprovincialis	Stress	8246	Peptidase inhibitor 16<br>Cardiac stress response	1.4	[4]
M. galloprovincialis	Shell formation	18160	EF-hand Ca2+-binding domain 6<br>CaLP has two Ca2+-binding EF hand domains.Growth of nacre-prismatic layer	1.2	[5]
M. galloprovincialis	Immunity	19933	Signal transducer-activator of transcription 5B<br>Mediate the signaling of cytokines and a number of growth factors	1.1	[6]
M. galloprovincialis	Stress	1594	Pyruvate dehydrogenase E1 alpha subunit<br>Reduces OXPHOS and oxygen consumption	1.1	[7]
M. galloprovincialis	Stress	973	DNA mismatch repair protein MSH6<br>Responsive to oxidative stress and protection against ROS and DNA damage	1.1	[8]
M. galloprovincialis	Stress	25447	zinc finger MYM-type protein 2-like<br>Significant correlations with salinity, temperature, As, Cd or lindane	1.1	[9]
M. galloprovincialis	Shell formation	12897	Ca2+ transporting ATPase, plasma membrane<br>Catalyse the hydrolysis of ATP coupled with the transport of calcium	1	[10]
M. galloprovincialis	Stress	14603	Inositol polyphosphate 1-phosphatase<br>Mg2+ dependent of inositol monophosphatase-like domain	1	[11]
M. galloprovincialis	Immunity	14873	Galectin-4<br>Regulators of immune cell homeostasis	1	[12]
M. galloprovincialis	Immunity	3134	PRRT1<br>Proline-rich transmembrane protein 1	1	[13]
M. galloprovincialis	Shell formation	14056	Metalloproteinase inhibitor 3<br>Ligament-specific protein	0.9	[14]
M. galloprovincialis	Stress	1893	Solute carrier family 12<br>Transport endogenous-exogenous substances. Potassium/chloride transporters	0.9	[15]
M. galloprovincialis	Immunity	7356	Leucine-rich repeat domain superfamily<br>Development, growth, and responses to abiotic and biotic stresses	0.9	[16]
M. galloprovincialis	Stress	3258	Heat shock protein 22 HSPB8<br>Protecting cells, folding of nascent peptides, and responding to stress	0.9	[17]
M. galloprovincialis	Stress	5345	Mitogen-activated protein kinase 6 MAPK<br>involved in the regulation of Hsp expression in blue mussels	0.9	[18]
M. galloprovincialis	Immunity	5490	LRP1B<br>Low-densitylipoproteinreceptor-related protein	0.9	[19]
M. edulis	Immunity	1359	E3 ubiquitin-protein ligase mind-bomb (MIB2)<br>Antiviral immunity	2.4	[20]
M. edulis	Stress	5390	Mucolipin, Polycystin cation channel<br>Calcium homeostasis	2	[21]
M. edulis	Immunity	13969	RNF170, RING finger protein 170<br>Ubiquitination and degradation of inositol 1,4,5-trisphosphate receptors	1.7	[22]
M. edulis	Stress	9109	Fibropellin-1<br>Hypoxia responsive gene	1.7	[23]
M. edulis	Stress	10101	Carbohydrate-binding WSC<br>Plasma membrane sensor for surface stress	1.5	[24]
M. edulis	Immunity	4324	Lactamase_B<br>Drug resistance among gram-negative bacteria	1.3	[25]
M. edulis	Stress	4307	polycystin<br>Calcium homeostasis	1.3	[26]
M. edulis	Immunity/stress	9741	Papain-like cysteine peptidase superfamily<br>Prevent unwanted protein degradation	1.2	[27]
M. edulis	Immunity	7225	Peptidase M12B<br>Cell adhesion, signaling, cell-cell fusion, and cell-cell interactions	1.2	[28]
M. edulis	Stress	7276	ATP-dependent metalloprotease (YME1L)<br>Stress-sensitive mitochondrial protease	1.2	[29]
M. edulis	Immunity	14877	P-loop - nucleoside triphosphate hydrolase<br>This domain shows a high specificity for pathogens and parasites	1.1	[30]
M. edulis	shell formation	15346	EF-hand Ca2+-binding domain<br>CaLP has two Ca2+-binding EF hand domains(Growth of nacre-prismatic layer)	1.1	[5]
M. edulis	Immunity	2105	C-type lectin superfamily 17 member A<br>Mediate crucial cellular functions during immunity and homeostasis	1	[31]
M. edulis	Immunity	10990	Fibrinogen-like protein A<br>Immunepattern-recognition receptors.	1	[32]
M. edulis	Immunity	20412	FYVE, RhoGEF and PH domain<br>Signal transduction	0.9	[33]
M. edulis	shell formation	5702	Perlucin-like protein<br>Ca2+-dependent carbohydrate binding activity	0.9	[34]
M. edulis	Immunity	22962	C1q-related factor<br>Pattern recognition receptors. Activates innate immune response	0.9	[35]
M. edulis	Immunity	11253	nicotinic acetylcholine receptor alpha-7<br>Regulates immune response through the neuroendocrine-immune system	0.9	[21]
M. edulis	Immunity	19695	TRIM56, tripartite motif-containing protein 56<br>virus-inducible E3 ubiquitin ligase that restricts pestivirus infection	0.9	[36]
M. edulis	Immunity	11784	HMCN, hemicentin<br>Immune recognition, signaling and regulation.insulin peptide receptor	0.9	[37]
M. edulis	shell formation	12528	COL6A, collagen, type VI<br>Adhesome molecules	0.9	[38]
M. edulis	shell formation	8193	mucin-13-like<br>Molluscan calcification	0.9	[13]
M. edulis	Stress	6739	serine-protein kinase ATM<br>DNA damage sensor	0.8	[39]
M. coruscus	Shell formation	8395	Hemicentin (HMCN)<br>Extracellular ion-binding proteins in the biomineral matrix	2	[40]
M. coruscus	Stress	2491	O-mannosyltransferase (TMTC)<br>Ca2+regulation and protein folding	2	[41]
M. coruscus	Immunity	5530	ADAR, adenosine deaminase<br>DNA binding. antiviral effectors	1.6	[42]
M. coruscus	Immunity	9626	Caveolin-1,Caveolin-3<br>Regulating neutrophil functional responses that underpin innate immunity	1.2	[43]
M. coruscus	Immunity	8044	Apoptosis regulator BAX<br>Apoptosis regulator	1.1	[44]
M. coruscus	Stress	17439	2-hydroxyglutaryl-CoA dehydratase (hgdC)<br>Iron-sulfur cluster binding	1.1	[45]
M. coruscus	Immunity	302	Mannose receptor, C type (MRC)<br>Pathogen recognition receptor	1	[20]
M. coruscus	Immunity	6467	Inhibitor of growth protein 1 (ING1)<br>Tumor suppressor gene	1	[46]
M. coruscus	Immunity	2098	BIRC2_3<br>Physiological role in growth, immunity, and apoptosis	1	[47]
M. coruscus	Immunity	8921	Filamin<br>Recognition of pathogens	1	[48]
M. coruscus	Immunity	13594	Proteasome regulatory (PSMD7)<br>Recognition of polyubiquitin chains and cleavage of ubiquitin from degraded proteins	0.9	[49]
M. coruscus	Shell formation	4996	NOTCH1<br>Calcium signalling pathway and shell pigmentation	0.9	[50]
M. coruscus	Stress	3709	Heat shock protein 90kDa beta (HSP90B)<br>Heat shock protein	0.9	[51]
M. coruscus	Immunity	8284	Cell division control protein 42<br>Roles in host defense	0.9	[52]
M. coruscus	Stress	5847	Ammonium transporter (amt)<br>Ammonium transporter	0.9	[53]
M. coruscus	Immunity	12429	RING finger protein 145<br>Ubiquitination	0.8	[54]
M. coruscus	Stress	12587	Lysine methyltransferase 4 (EEF1AKMT4)<br>Enhances the function of heat shock factor 1 during the heat shock response	0.8	[55]

Author details
Institute of Aquaculture, Faculty of Natural Sciences, University of Stirling, Stirling, FK9 4LA UK.

References

1. Lin, C., Zhang, J., Lu, Y., Li, X., Zhang, W., Zhang, W., Lin, W., Zheng, L., Li, X.: NIT1 suppresses tumour proliferation by activating the TGFβ1-Smad2/3 signalling pathway in colorectal cancer. Cell death & disease **9**(3), 263 (2018). doi:10.1038/s41419-018-0333-3
2. Bonacci, S., Browne, M.A., Dissanayake, A., Hagger, J.A., Corsi, I., Focardi, S., Galloway, T.S.: Esterase activities in the bivalve mollusc *Adamussium colbecki* as a biomarker for pollution monitoring in the antarctic marine environment. Marine Pollution Bulletin **49**(5-6), 445–455 (2004). doi:10.1016/j.marpolbul.2004.02.033
3. Zorina, A.A., Bedbenov, V.S., Novikova, G.V., Panichkin, V.B., Los', D.A.: Involvement of serine/threonine protein kinases in the cold stress response in the cyanobacterium *Synechocystis* sp. PCC 6803: Functional characterization of SpkE protein kinase. Molecular Biology **48**(3), 390–398 (2014). doi:10.1134/S0026893314030212
4. Regn, M., Laggerbauer, B., Jentzsch, C., Ramanujam, D., Ahles, A., Sichler, S., Calzada-Wack, J., Koenen, R.R., Braun, A., Nieswandt, B., Engelhardt, S.: Peptidase inhibitor 16 is a membrane-tethered regulator of chemerin processing in the myocardium. Journal of Molecular and Cellular Cardiology **99**, 57–64 (2016). doi:10.1016/j.yjmcc.2016.08.010
5. Feng, D., Li, Q., Yu, H., Kong, L., Du, S.: Identification of conserved proteins from diverse shell matrix proteome in *Crassostrea gigas*: Characterization of genetic bases regulating shell formation. Scientific Reports **7**(April), 1–12 (2017). doi:10.1038/srep45754
6. Yu, M., Zheng, L., Wang, X., Wu, M., Qi, M., Fu, W., Zhang, Y.: Comparative transcriptomic analysis of surf clams (*Paphia undulate*) infected with two strains of *Vibrio* spp. reveals the identity of key immune genes involved in host defense. BMC Genomics **20**(1), 988 (2019). doi:10.1186/s12864-019-6351-4
7. Zimmer, A.D., Walbrecq, G., Kozar, I., Behrmann, I., Haan, C.: Phosphorylation of the pyruvate dehydrogenase complex precedes HIF-1-mediated effects and pyruvate dehydrogenase kinase 1 upregulation during the first hours of hypoxic treatment in hepatocellular carcinoma cells. Hypoxia **4**, 135–145 (2016). doi:10.2147/HP.S99044
8. Pinheiro, M., Oliveira, A., Barros, S., Alves, N., Raimundo, J., Caetano, M., Coimbra, J., Neuparth, T., Santos, M.M.: Functional, biochemical and molecular impact of sediment plumes from deep-sea mining on *Mytilus galloprovincialis* under hyperbaric conditions. Environmental research **195**, 110753 (2021). doi:10.1016/j.envres.2021.110753
9. Baillon, L., Pierron, F., Coudret, R., Normendeau, E., Caron, A., Peluhet, L., Labadie, P., Budzinski, H., Durrieu, G., Sarraco, J., Elie, P., Couture, P., Baudrimont, M., Bernatchez, L.: Transcriptome profile analysis reveals specific signatures of pollutants in Atlantic eels. Ecotoxicology (London, England) **24**(1), 71–84 (2015). doi:10.1007/s10646-014-1356-x
10. Sillanpää, J.K., Sundh, H., Sundell, K.S.: Calcium transfer across the outer mantle epithelium in the pacific oyster, *Crassostrea gigas*. Proceedings of the Royal Society B: Biological Sciences **285**(1891), 20181676 (2018). doi:10.1098/rspb.2018.1676
11. Bialojan, C., Takai, A.: Inhibitory effect of a marine-sponge toxin, okadaic acid, on protein phosphatases. specificity and kinetics. Biochemical Journal **256**(1), 283–290 (1988). doi:10.1042/bj2560283
12. Vasta, G.R., Feng, C., Bianchet, M.A., Bachvaroff, T.R., Tasumi, S.: Structural, functional, and evolutionary aspects of galectins in aquatic mollusks: From a sweet tooth to the Trojan horse. Fish & Shellfish Immunology **46**(1), 94–106 (2015). doi:10.1016/j.fsi.2015.05.012
13. Marin, F., Corstjens, P., de Gaulejac, B., de Vrind-De Jong, E., Westbroek, P.: Mucins and molluscan calcification. Molecular characterization of mucoperlin, a novel mucin-like protein from the nacreous shell layer of the fan mussel *Pinna nobilis* (Bivalvia, pteriomorphia). The Journal of biological chemistry **275**(27), 20667–75 (2000). doi:10.1074/jbc.M003006200
14. Kubota, K., Tsuchihashi, Y., Kogure, T., Maeyama, K., Hattori, F., Kinoshita, S., Sakuda, S., Nagasawa, H., Yoshimura, E., Suzuki, M.: Structural and functional analyses of a TIMP and MMP in the ligament of *Pinctada fucata*. Journal of Structural Biology **199**(3), 216–224 (2017). doi:10.1016/j.jsb.2017.07.010
15. Xun, X., Cheng, J., Wang, J., Li, Y., Li, X., Li, M., Lou, J., Kong, Y., Bao, Z., Hu, X.: Solute carriers in scallop genome: Gene expansion and expression regulation after exposure to toxic dinoflagellate. Chemosphere **241**, 124968 (2020). doi:10.1016/j.chemosphere.2019.124968
16. Wang, X., Wang, M., Xu, Q., Xu, J., Lv, Z., Wang, L., Song, L.: Two novel LRR and Igdomain-containing proteins from oyster *Crassostrea gigas* function as pattern recognition receptors and induce expression of cytokines. Fish & Shellfish Immunology **70**, 308–318 (2017). doi:10.1016/j.fsi.2017.09.023
17. Zhang, L., Lingling, A.E., Ae, W., Song, L., Jianmin, A.E., Ae, Z., Qiu, L., Chaohua, A.E., Ae, D., Li, F., Huan, A.E., Ae, Z., Yang, G.: The involvement of HSP22 from bay scallop *Argopecten irradians* in response to heavy metal stress. Molecular biology reports **37**(4), 1763–71 (2010). doi:10.1007/s11033-009-9603-6
18. Anestis, A., Lazou, A., Pörtner, H.O., Michaelidis, B.: Behavioral, metabolic, and molecular stress responses of marine bivalve *Mytilus galloprovincialis* during long-term acclimation at increasing ambient temperature. American Journal of Physiology-Regulatory, Integrative and Comparative Physiology **293**(2), 911–921 (2007). doi:10.1152/ajpregu.00124.2007
19. Liu, R., Wang, L., Sun, Y., Wang, L., Zhang, H., Song, L.: A low-density lipoprotein receptor-related protein (LRP)-like molecule identified from *Chlamys farreri* participated in immune response against bacterial infection. Fish and Shellfish Immunology **36**(2), 336–343 (2014). doi:10.1016/j.fsi.2013.11.017
20. Chen, H., Wang, L., Zhou, Z., Hou, Z., Liu, Z., Wang, W., Gao, D., Gao, Q., Wang, M., Song, L.: The comprehensive immunomodulation of neurimmirs in haemocytes of oyster *Crassostrea gigas* after acetylcholine and norepinephrine stimulation. BMC Genomics **16**(1), 1–14 (2015). doi:10.1186/s12864-015-2150-8
21. Jiao, Y., Cao, Y., Zheng, Z., Liu, M., Guo, X.: Massive expansion and diversity of nicotinic acetylcholine receptors in lophotrochozoans. BMC Genomics **20**(1), 937 (2019). doi:10.1186/s12864-019-6278-9

22. Song, X., Liu, Z., Wang, L., Song, L.: Recent advances of shell matrix proteins and cellular orchestration in marine molluscan shell biomineralization. Frontiers in Marine Science **6**(FEB) (2019). doi:10.3389/fmars.2019.00041
23. Nie, H., Wang, H., Jiang, K., Yan, X.: Transcriptome analysis reveals differential immune related genes expression in *Ruditapes philippinarum* under hypoxia stress: potential HIF and NF-κB crosstalk in immune responses in clam. BMC Genomics **21**(1), 318 (2020). doi:10.1186/s12864-020-6734-6
24. Oide, S., Tanaka, Y., Watanabe, A., Inui, M.: Carbohydrate-binding property of a cell wall integrity and stress response component (WSC) domain of an alcohol oxidase from the rice blast pathogen *Pyricularia oryzae*. Enzyme and Microbial Technology **125**, 13–20 (2019). doi:10.1016/j.enzmictec.2019.02.009
25. Singh, T., Singh, P.K., Das, S., Wani, S., Jawed, A., Dar, S.A.: Transcriptome analysis of beta-lactamase genes in diarrheagenic *Escherichia coli*. Scientific Reports **9**(1), 3626 (2019). doi:10.1038/s41598-019-40279-1
26. Wang, X., Wang, M., Wang, W., Liu, Z., Xu, J., Jia, Z., Chen, H., Qiu, L., Lv, Z., Wang, L., Song, L.: Transcriptional changes of pacific oyster *Crassostrea gigas* reveal essential role of calcium signal pathway in response to co_2-driven acidification. Science of The Total Environment **741**, 140177 (2020). doi:10.1016/j.scitotenv.2020.140177
27. Liu, H., Hu, M., Wang, Q., Cheng, L., Zhang, Z.: Role of Papain-like cysteine proteases in plant development. Frontiers in Plant Science **9**, 1717 (2018). doi:10.3389/fpls.2018.01717
28. Rubin, E., Tanguy, A., Perrigault, M., Pales Espinosa, E., Allam, B.: Characterization of the transcriptome and temperature-induced differential gene expression in QPX, the thraustochytrid parasite of hard clams. BMC Genomics **15**(1), 1–16 (2014). doi:10.1186/1471-2164-15-245
29. Rainbolt, T.K., Saunders, J.M., Wiseman, R.L.: YME 1L degradation reduces mitochondrial proteolytic capacity during oxidative stress. EMBO reports **16**(1), 97–106 (2015). doi:10.15252/embr.201438976
30. Arivalagan, J., Yarra, T., Marie, B., Sleight, V.A., Duvernois-Berthet, E., Clark, M.S., Marie, A., Berland, S.: Insights from the shell proteome: Biomineralization to adaptation. Molecular biology and evolution **34**(1), 66–77 (2017). doi:10.1093/molbev/msw219
31. Kerscher, B., Willment, J.A., Brown, G.D.: The Dectin-2 family of C-type lectin-like receptors: an update. International immunology **25**(5), 271–7 (2013). doi:10.1093/intimm/dxt006
32. Gorbushin, A.M., Iakovleva, N.V.: A new gene family of single fibrinogen domain lectins in *Mytilus*. Fish & shellfish immunology **30**(1), 434–8 (2011). doi:10.1016/j.fsi.2010.10.002
33. Perrier, F., Bertucci, A., Pierron, F., Feurtet-Mazel, A., Simon, O., Klopp, C., Candaudap, F., Pokrovski, O., Etcheverria, B., Mornet, S., Baudrimont, M.: Transfer and transcriptomic profiling in liver and brain of european eels (*Anguilla anguilla*) after diet-borne exposure to gold nanoparticles. Environmental toxicology and chemistry **39**(12), 2450–2461 (2020). doi:10.1002/etc.4858
34. Blank, S., Arnoldi, M., Khoshnavaz, S., Treccani, L., Kuntz, M., Mann, K., Grathwohl, G., Fritz, M.: The nacre protein perlucin nucleates growth of calcium carbonate crystals. Journal of Microscopy **212**(3), 280–291 (2003). doi:10.1111/j.1365-2818.2003.01263.x
35. Jiang, K., Nie, H., Li, D., Yan, X.: New insights into the Manila clam and PAMPs interaction based on RNA-seq analysis of clam through *in vitro* challenges with LPS, PGN, and poly(I:C). BMC Genomics **21**(1), 1–18 (2020). doi:10.1186/s12864-020-06914-2
36. Liu, B., Li, N.L., Wang, J., Shi, P.-Y., Wang, T., Miller, M.A., Li, K.: Overlapping and distinct molecular determinants dictating the antiviral activities of TRIM56 against flaviviruses and coronavirus. Journal of virology **88**(23), 13821–35 (2014). doi:10.1128/JVI.02505-14
37. Wang, K., del Castillo, C., Corre, E., Pales Espinosa, E., Allam, B.: Clam focal and systemic immune responses to QPX infection revealed by RNA-seq technology. BMC Genomics **17**(1), 146 (2016). doi:10.1186/s12864-016-2493-9
38. Dyachuk, V.: Extracellular matrix components in Bivalvia: Shell and ECM components in developmental and adult tissues. Fisheries and Aquaculture Journal **9**(2) (2018). doi:10.4172/2150-3508.1000248
39. Matsuoka, M., Igisu, H.: Cadmium induces phosphorylation of p53 at serine 15 in MCF-7 cells. Biochemical and biophysical research communications **282**(5), 1120–5 (2001). doi:10.1006/bbrc.2001.4700
40. Luo, Y.-J., Takeuchi, T., Koyanagi, R., Yamada, L., Kanda, M., Khalturina, M., Fujie, M., Yamasaki, S.-I., Endo, K., Satoh, N.: The lingula genome provides insights into brachiopod evolution and the origin of phosphate biomineralization. Nature Communications **6**(1), 8301 (2015). doi:10.1038/ncomms9301
41. Larsen, I.S.B., Narimatsu, Y., Joshi, H.J., Siukstaite, L., Harrison, O.J., Brasch, J., Goodman, K.M., Hansen, L., Shapiro, L., Honig, B., Vakhrushev, S.Y., Clausen, H., Halim, A.: Discovery of an O-mannosylation pathway selectively serving cadherins and protocadherins. Proceedings of the National Academy of Sciences of the United States of America **114**(42), 11163–11168 (2017). doi:10.1073/pnas.1708319114
42. Green, T.J., Rolland, J.-L., Vergnes, A., Raftos, D., Montagnani, C.: OsHV-1 countermeasures to the Pacific oyster's anti-viral response. Fish & shellfish immunology **47**(1), 435–43 (2015). doi:10.1016/j.fsi.2015.09.025
43. Zemans, R., Downey, G.P.: Role of caveolin-1 in regulation of inflammation: different strokes for different folks. American Journal of Physiology-Lung Cellular and Molecular Physiology **294**(2), 175–177 (2008). doi:10.1152/ajplung.00488.2007
44. Leprêtre, M., Almunia, C., Armengaud, J., Le Guernic, A., Salvador, A., Geffard, A., Palos-Ladeiro, M.: Identification of immune-related proteins of *Dreissena polymorpha* hemocytes and plasma involved in host-microbe interactions by differential proteomics. Scientific reports **10**(1), 6226 (2020). doi:10.1038/s41598-020-63321-z
45. Locher, K.P., Hans, M., Yeh, A.P., Schmid, B., Buckel, W., Rees, D.C.: Crystal structure of the *Acidaminococcus fermentans* 2-hydroxyglutaryl-CoA dehydratase component A. Journal of Molecular Biology **307**(1), 297–308 (2001). doi:10.1006/jmbi.2000.4496
46. Garkavtsev, I., Grigorian, I.A., Ossovskaya, V.S., Chernov, M.V., Chumakov, P.M., Gudkov, A.V.: The candidate tumour suppressor p33ING1 cooperates with p53 in cell growth control. Nature **391**(6664), 295–298 (1998). doi:10.1038/34675
47. Wilson, J.J., Grendler, J., Dunlap-Smith, A., Beal, B.F., Page, S.T.: Analysis of gene expression in an inbred

line of soft-shell clams (*Mya arenaria*) displaying growth heterosis: Regulation of structural genes and the NOD2 pathway. International journal of genomics **2016**, 6720947 (2016). doi:10.1155/2016/6720947

48. Maldonado-Aguayo, W., Lafarga-De la Cruz, F., Gallardo-Escárate, C.: Identification and expression of antioxidant and immune defense genes in the surf clam *Mesodesma donacium* challenged with *Vibrio anguillarum*. Marine Genomics **19**, 65–73 (2015). doi:10.1016/j.margen.2014.11.006
49. Smits, M., Artigaud, S., Bernay, B., Pichereau, V., Bargelloni, L., Paillard, C.: A proteomic study of resistance to Brown Ring disease in the manila clam, *Ruditapes philippinarum*. Fish & Shellfish Immunology **99**, 641–653 (2020). doi:10.1016/j.fsi.2020.02.002
50. Auffret, P., Le Luyer, J., Sham Koua, M., Quillien, V., Ky, C.-L.: Tracing key genes associated with the *Pinctada margaritifera* albino phenotype from juvenile to cultured pearl harvest stages using multiple whole transcriptome sequencing. BMC Genomics **21**(1), 662 (2020). doi:10.1186/s12864-020-07015-w
51. Cao, R., Wang, D., Wei, Q., Wang, Q., Yang, D., Liu, H., Dong, Z., Zhang, X., Zhang, Q., Zhao, J.: Integrative biomarker assessment of the influence of saxitoxin on marine bivalves: A comparative study of the two bivalve species oysters, *Crassostrea gigas*, and scallops, *Chlamys farrer*. Frontiers in physiology **9**(AUG), 1173 (2018). doi:10.3389/fphys.2018.01173
52. Xu, J.-D., Jiang, H.-S., Wei, T.-D., Zhang, K.-Y., Wang, X.-W., Zhao, X.-F., Wang, J.-X.: Interaction of the small GTPase cdc42 with arginine kinase restricts White spot syndrome virus in shrimp. Journal of Virology **91**(5) (2017). doi:10.1128/jvi.01916-16
53. Bu, Y., Takano, T., Liu, S.: The role of ammonium transporter (AMT) against salt stress in plants. Plant signaling & behavior **14**(8), 1625696 (2019). doi:10.1080/15592324.2019.1625696
54. Cook, E.C.L., Nelson, J.K., Sorrentino, V., Koenis, D., Moeton, M., Scheij, S., Ottenhoff, R., Bleijlevens, B., Loregger, A., Zelcer, N.: Identification of the ER-resident E3 ubiquitin ligase RNF145 as a novel LXR-regulated gene. PLoS One **12**(2), 0172721 (2017). doi:10.1371/journal.pone.0172721
55. Vera, M., Pani, B., Griffiths, L.A., Muchardt, C., Abbott, C.M., Singer, R.H., Nudler, E.: The translation elongation factor eef1a1 couples transcription to translation during heat shock response. eLife **3**, 03164 (2014). doi:10.7554/eLife.03164
